# Regioselective Radical Reaction of Monometallofullerene Y@C_2v_(9)-C_82_ With N-arylbenzamidine Mediated by Silver Carbonate

**DOI:** 10.3389/fchem.2020.593602

**Published:** 2020-10-20

**Authors:** Jia Li, Pengyuan Yu, Peng Lai, Jiajun Zou, Zhe Liu, Xiuguang Yi, Wei Wang, Changwang Pan

**Affiliations:** ^1^School of Chemistry and Chemical Engineering, Jinggangshan University, Ji'an, China; ^2^State Key Laboratory of Materials Processing and Die & Mold Technology, School of Materials Science and Engineering, Huazhong University of Science and Technology, Wuhan, China

**Keywords:** metallofullerene, radical reaction, silver carbonate, functionalization, imidazoline

## Abstract

A novel radical reaction of monometallofullerene Y@C_2v_(9)-C_82_ with N-arylbezamidine (**1**) is successfully conducted through catalysis with silver carbonate. The high-performance liquid chromatographic and mass spectrum results demonstrate that the reaction is highly regioselective to afford only one monoadduct (**2**) with an imidazoline group added on C_82_ cage, and computations through density functional theory reveal the addition group is attached to a specific [5, 6]-bond (C20-C76) near the Y atom. Furthermore, the analysis of prymidalization angle of the carbon atoms demonstrates the geometry of carbon cage is in favor of the regioselective formation of isomer (20, 76).

## Introduction

Exohedral chemical functionalization of fullerenes has a great significance toward their potential applications in photovoltaic and biomedical fields. To date, a large number of reactions, including but not limit to Bingle–Hirsch reactions, Prato reactions, Diels–Alder reactions, and radical reactions, have been successfully performed to modulate their chemical and physical properties (Hirsch and Brettreich, 2005). Among them, of particular interest are the radical reactions of fullerenes mediated by transition-metal salts (Tzirakis and Orfanopoulos, 2013), and many catalyzers, such as Mn(OAc)_3_, Fe(ClO_4_)_3_ (Zhang et al., 2003; Wang et al., 2004; Li et al., 2010a,b, 2012; Liu et al., 2011), Pb(OAc)_4_ (Chai and Lautens, 2009), Cu(OAc)_2_ (Wang and Li, 2005), CoCl_2_ (Lu S. et al., 2011), NiCl_2_ (Constable et al., 2012), CuCl_2_ (Yang et al., 2013; Sharma et al., 2018), and FeCl_3_ (Hashiguchi et al., 2013, 2014), have been utilized to produce radicals of fullerenes to promote the radical reactions taking advantage of their excellent catalytic activities. Because of the high efficiency in constructing multiple new chemical bonds, thus leading to derivatives with various structures in one step, this class of reactions becomes more and more important in functionalization of fullerenes.

On the other hand, endohedral metal doping of the fullerene cages can generate a novel class of hybrid molecules named endohedral metallofullerenes (EMFs) (Lu et al., 2012; Popov et al., 2013; Yang et al., 2017; Bao et al., 2018). The interaction between the internal metallic unit and fullerene carbon cages renders the chemistry of EMFs more complicated but also intriguing compared to that of empty fullerenes (Lu X. et al., 2011; Maeda et al., 2016). As a result, the amount of metal-mediated reactions of EMFs lags far behind those of fullerenes and the regioselectivity is just satisfactory with the generation of two or more isomers. Typically, Gu et al. synthesize a series of water-soluble multiadducts of Tb@C_82_ with Cu(MeCN)_4_PF_6_ as a catalyst in 2002 (Feng et al., 2002), and subsequently Dorn et al. utilized manganese(III) acetate to trigger the radical reaction of Sc_3_N@C_80_ and obtained two isomers methano monoadducts in 2007 (Feng et al., 2002; Shu et al., 2007). The rare reports about this kind of reactions can be understood by considering the direct reaction of EMFs with metallic salts such as CuCl_2_, NiCl_2_, and FeCl_3_, which forms solid precipitate instead of target reactants (Stevenson et al., 2009, 2014; Stevenson and Rottinger, 2013; Wang et al., 2017), and thus it is still of high interest and challenge to seek for the catalyst with appropriate activity and the regioselectivity for the metal-mediated reactions of EMFs.

In this work, we found that silver carbonate is an efficient catalyst to promote the reaction of Y@C_2v_(9)-C_82_ with N-arylbenzamidine. Remarkably, the reaction regioselectively affords only one derivative as revealed by high-performance liquid chromatography (HPLC) and mass spectrometry (MS), and the density functional theory calculations predict the addition is preferentially occurred on a specific [5, 6]-bond with large prymidalization angle near the Y atom.

## Materials and Methods

The solvent toluene was freshly distilled with sodium prior to usage. The reagent Ag_2_CO_3_ was obtained commercially, and N-arylbenzamidine was synthesized according to a previous report, and the structure was determined through ^1^H NMR (Supplementary Figure 5) (Koutentis and Mirallai, 2010). Y@C_2v_(9)-C_82_ was produced with arc-discharge method and isolated with HPLC. Analytical and preparative HPLC measurements were conducted on LC SPD-16 and LC 908 machines (Japan Analytical Industry Co., Ltd.), respectively. Matrix-assisted laser desorption/ionization time-of-flight (MALDI-TOF) MS was measured on a MICROFLEX spectrometer (Bruker Daltonics Inc., Germany), using 1,1,4,4-tetraphenyl-1,3-butadiene as matrix in a positive ion linear mode.

Y@C_2v_(9)-C_82_ and all possible isomers formed by the addition of **1** on non-equivalent C-C bonds, were constructed and optimized at HF level of theory with 3-21G for C, H, and N atoms and LANL2DZ for Y. Among them, low-lying isomers were chosen out and reoptimized using B3LYP (Lee et al., 1988; Becke, 1993) functional with 6-31G(d) for non-metals and SDD for Y. All computations were performed with Gaussian 09 Program (Frisch et al., 2013), and numbering of carbons in C_2v_(9)-C_82_ cage was given in Supplementary Figure 1 according to CAGE code (Brinkmann et al., 2010).

## Results and Discussion

**Figure 1:**
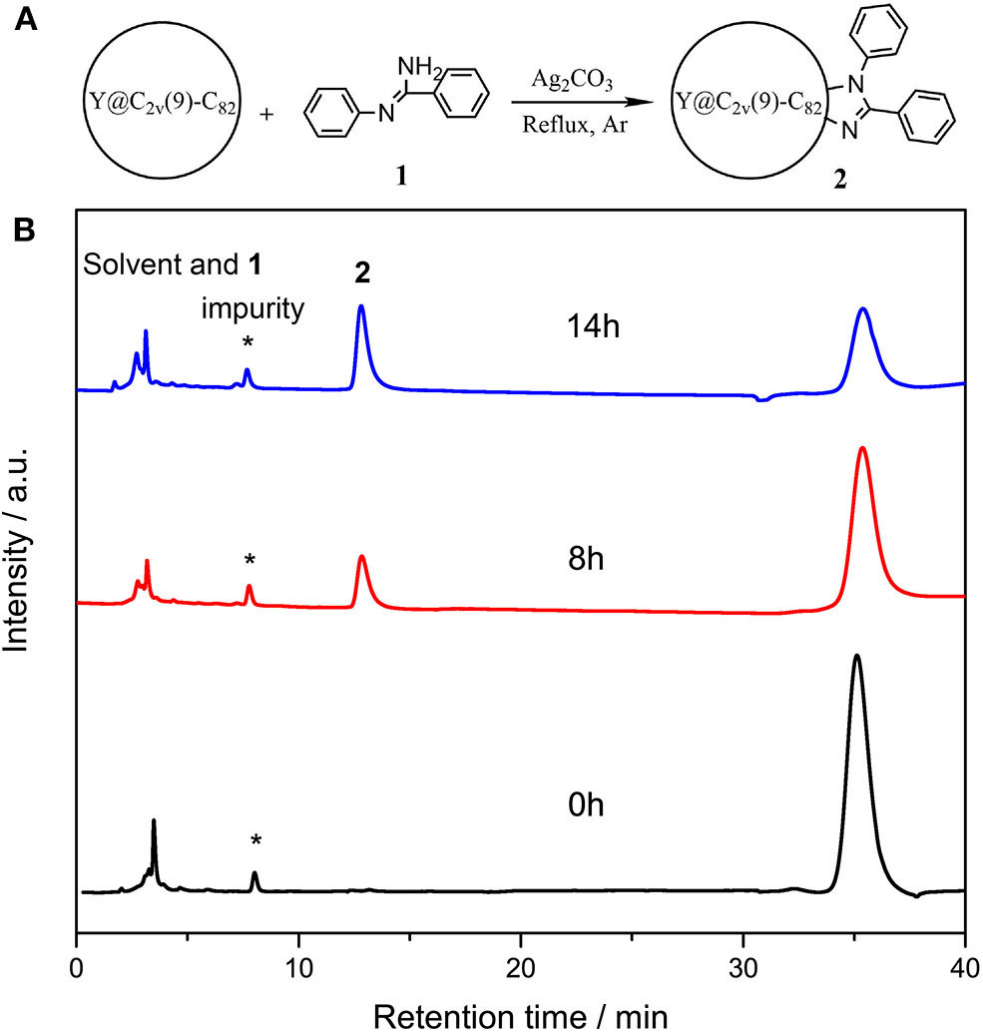
**(A)** Scheme of the reaction o of Y@C_2v_(9)-C_82_ with **1** and **(B)** analytical HPLC profiles of the reaction mixture of Y@C_2v_(9)-C_82_ and 1 probed at different time. HPLC condition: Buckyprep column (Φ4.6 mm × 250 mm); 20 μL injection volume; 1.0 mL/min toluene flow; room temperature; 330 nm detecting wavelength.

In a typical reaction, 5.0 mg (4.7 μmol) of Y@C_2v_(9)-C_82_ and 18.4 mg (20 eq) of N-arylbenzamidine (**1**) were dissolved in 25 mL of anhydrous toluene, and 13.0 mg (10 eq) of Ag_2_CO_3_ was added into the solution, and then the mixture was heated at reflux under argon (Figure 1A). The reaction process was monitored through analytical HPLC, and the profiles are shown in Figure 1B. At the beginning of the reaction, two peaks corresponding to the solvent and pristine metallofullerene were detected at 3.6 and 35.4 min, respectively. A new peak at 12.9 min increased along with the decreasing amount of metallofullerene, and the reaction was terminated because the peak almost keeps constant after 12 h. The above HPLC results demonstrate that the reaction possesses moderate reactivity and high selectivity. Besides, some silver salts such as silver nitrate, silver acetate, and silver trifluoroacetate, were applied to replace the catalyst silver carbonate; however, the results monitored by HPLC show no product was detected, but decreasing amount of the pristine metallofullerene, which is probably because Y@C_2v_(9)-C_82_ directly reacts and forms precipitates with these salts, impeding their mediated reaction between metallofullerene and **1**.

**Figure 2:**
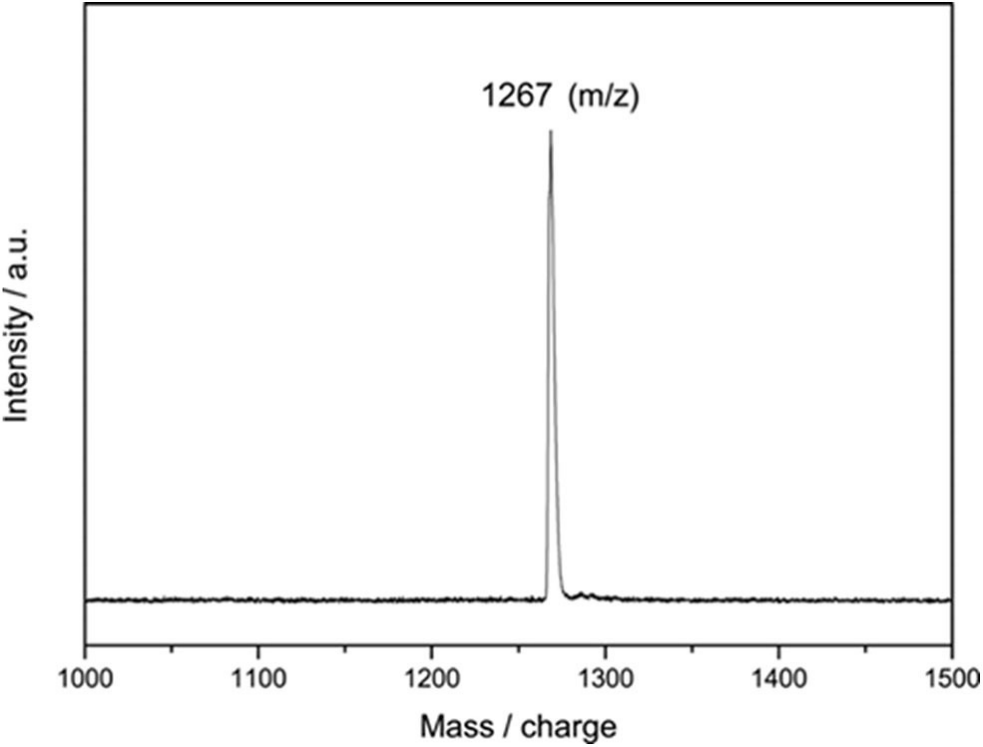
MALDI-TOF mass spectrum of product **2**.

The reaction mixture was then concentrated and subjected to further HPLC separations (Supplementary Figure 1), and 3.4 mg of pure compound **2** as black solids was obtained, and 2 mg of Y@C_2v_(9)-C_82_ was recollected. A large portion (up to 95%) of consumed EMF was converted to **2**, even much excess amounts of **1** were added, indicating the high regioselectivity of this catalytic reaction. The purified **2** is characterized through the MALDI-TOF MS, and the result shows only one peak at m/z 1267 was detected (Figure 2). It demonstrates that a group with 194 of molecular weight was added on the carbon cage, which is similar to the results of the reaction of C_60_ with **1** affording an imidazoline monoadduct (He et al., 2013). Consequently, it is speculated that **2** should be an imidazoline monoadduct too.

**Figure 3:**
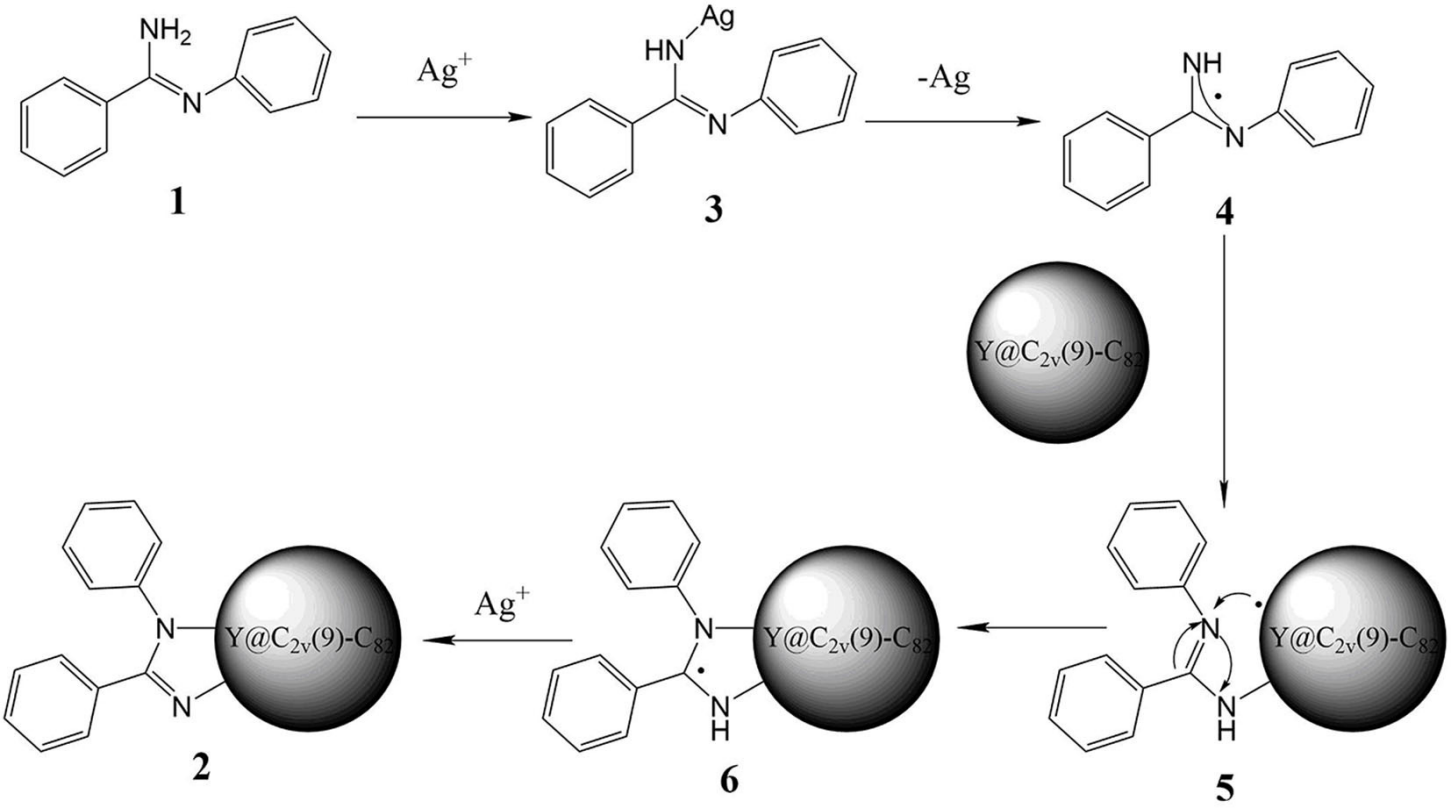
Possible mechanism of the reaction between Y@C_2v_(9)-C_82_ and **1**.

Based on the above experimental results and previous reports about metal-mediated fullerene reactions (He et al., 2013; Aghabali et al., 2016; Chao et al., 2016), a plausible reaction mechanism for this silver carbonate-catalyzed reaction is proposed in Figure 3. At first, N-arylbenzimidamide **1** directly reacts with Ag^+^, which generates **3**, and then a radical species **4** is obtained through homolytic cleavage of the nitrogen silver bond. Second, the radical addition of Y@C_2v_(9)-C_82_ will produce intermediate fullerenyl radical **5**, which may also be formed by the homolytic addition of **3** to metalofullerene, and then the intramolecular cyclization of **5** produces radical species **6**. Finally, the intermediate **6** is oxidated by Ag^+^ and loses the extra H^+^, affording the imidazoline derivative **2**.

**Figure 4:**
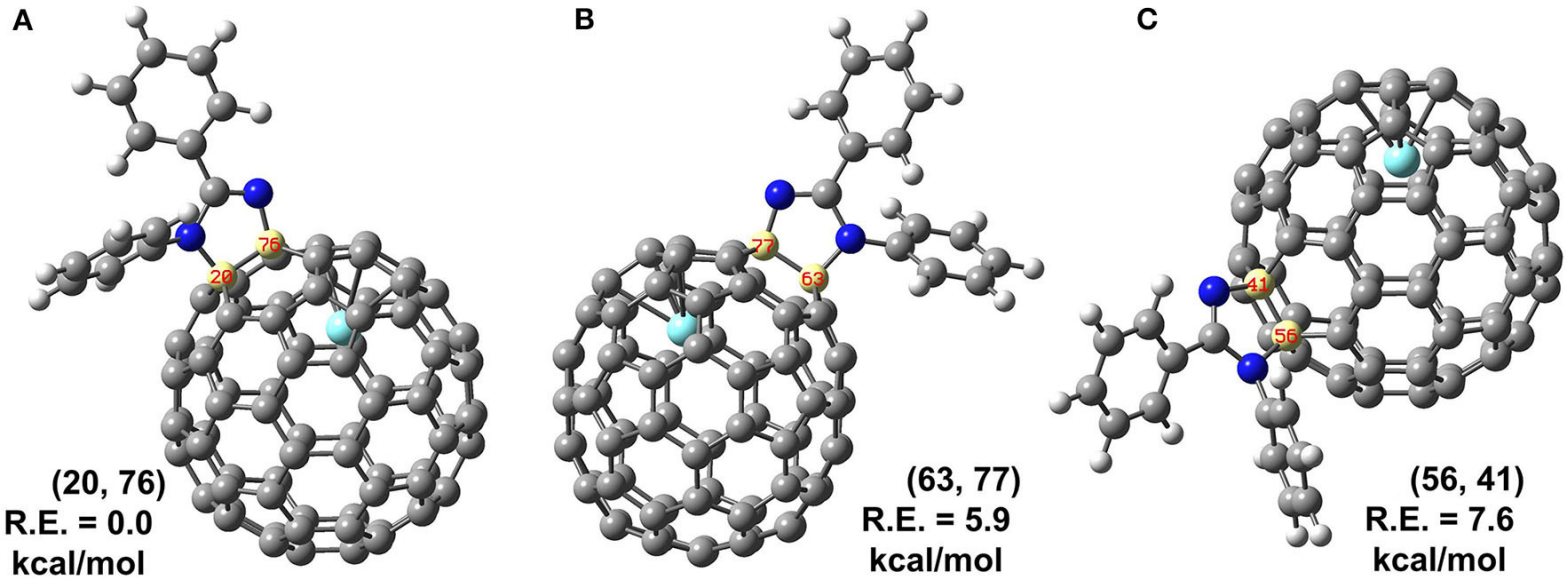
**(A–C)** Structures and relative energies (R.E., in kcal/mol) of isomers (20, 76), (63, 77) and (56, 41) predicated by B3LYP method. Isomers are labeled by a pair of numbers to indicate which carbons are attached by **1**.

Theoretical calculations were conducted to further determine the accurate structure and addition site of **2**. There are in total 35 non-equivalent bonds in the cage when *C*_2v_ symmetry was taken into consideration, and all the corresponding isomers of **2** named according to the addition bonds were optimized, and the relative energies are given in Supplementary Table 1. Three low-lying isomers were reoptimized using B3LYP functional with SDD basis for Y and 6-31G(d) for non-metal atoms; more accurate relative energies are obtained and given in Figure 4. The results show the energy of isomer (20, 76) is far lower than those of other isomers (63, 77) and (56, 41) with 5.9 and 7.6 kcal/mol, respectively, indicating the reaction should prefer to occur at the [5, 6]-bond near the internal metal atom. Besides relative energies, stability of isomer (20, 76) can also be disclosed by inspection on its spin density and singly occupied molecular orbital (SOMO), which show the spin density and SOMO of isomer (20, 76) are not concentrated on some specific carbons but evenly distributed over the whole cage (Supplementary Figure 3).

**Figure 5:**
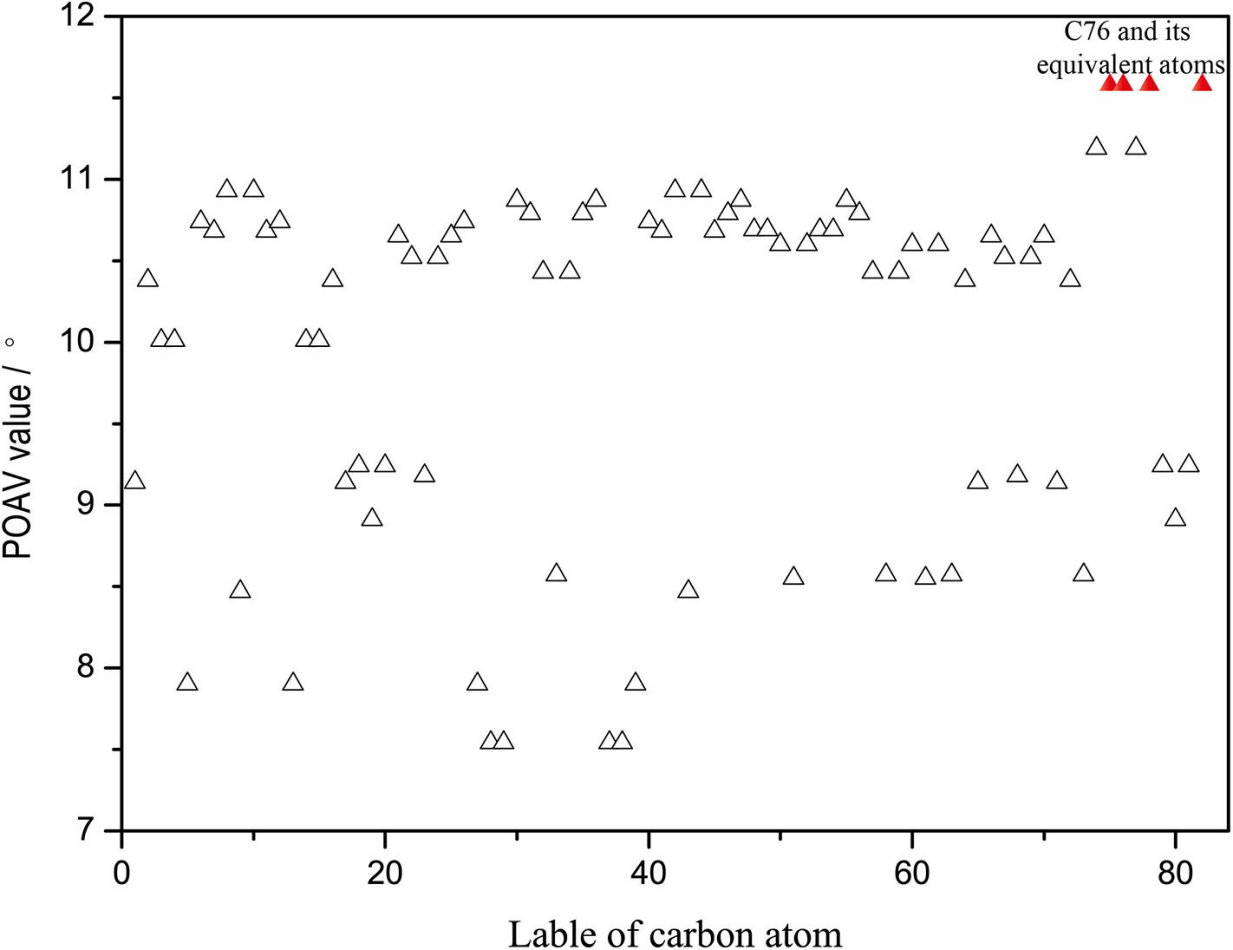
The POAV values of the carbon atoms on the C_2v_(9)-C_82_ cage.

Furthermore, the kinetic stability of isomer (20, 76) can also be rationalized from the pyramidalization angles of carbon atoms on the C_2v_(9)-*C*_82_ cage, which are strongly dependent on the cage geometry. In general, the addition reactions of fullerenes preferentially occur at the carbon atoms with relative high spin population and/or large POAV [the p-orbital axis vector (θ_Δπ_ − 90°)] values. The B3LYP predicated spin density of Y@C_2v_(9)-*C*_82_ and spin population condensed on each carbon atom are given in Supplementary Figure 4. The spin density distributed over the cage quite evenly and spin population of carbon atoms are also relatively small (ranges from −0.027 to 0.082), which is in agreement with the previous report (Bao et al., 2016). As a consequence, there are no carbon atoms of Y@C_2v_(9)-*C*_82_ possessing the distinct advantage of radical to regioselectively react with **1**. In contrast, as can be seen in Figure 5, the carbon atoms located near the Y atom possess evidently higher POAV values than others. In fact, the carbon atom C76 has largest POAV value up to 11.6°, and thus, C76 is certainly more reactive than other cage carbons to release its steric strain. After linking with C76, the adjacent carbon atom C20 has higher POAV value (9.7) than the other two adjacent carbon atoms C75 (5.3) and C77 (5.3), and thus, the addition group makes a second bond with C20 to form a [5, 6] monoadduct. Accordingly, the above results reveal the geometry of the carbon cage plays a role on the regioselective formation of the isomer (20, 76).

## Conclusion

In summary, a novel derivative **2** of monometallofullerene was synthesized via a highly regioselective reaction of **1** catalyzed by silver carbonate. Studies of MS and theoretical calculations disclose that an imidazoline group is attached to [5, 6]-bond near the metal atom forming a monoadduct. Additionally, the analysis of POAV values on carbon cages demonstrates that the geometry of carbon cage is conductive to regioselectively afford the isomer (20, 76). We believe the successful functionalization of metallofullerene mediated by transition metal will broaden the approach to chemistry of EMFs, which may find applications in photovoltaic and biomedical fields.

## Data Availability Statement

All datasets generated for this study are included in the article/Supplementary Material.

## Author Contributions

Experiments conceived and designed and the paper written by CP. Experiments and calculations performed by CP, JL, and PY. WW, XY, PL, JZ, and ZL analyzed the data. All authors contributed to the article and approved the submitted version.

## Conflict of Interest

The authors declare that the research was conducted in the absence of any commercial or financial relationships that could be construed as a potential conflict of interest.
