# Supplementary material for: Regioselective Radical Reaction of Monometallofullerene Y@C2v(9)-C82 With N-arylbenzamidine Mediated by Silver Carbonate
Source: Front Chem. 2020 Oct 20;8:593602. doi: 10.3389/fchem.2020.593602 (PMC7606928; doi:10.3389/fchem.2020.593602)
Supplement: Supplementary file 1 [file Data_Sheet_1.pdf]

Supplementary Material

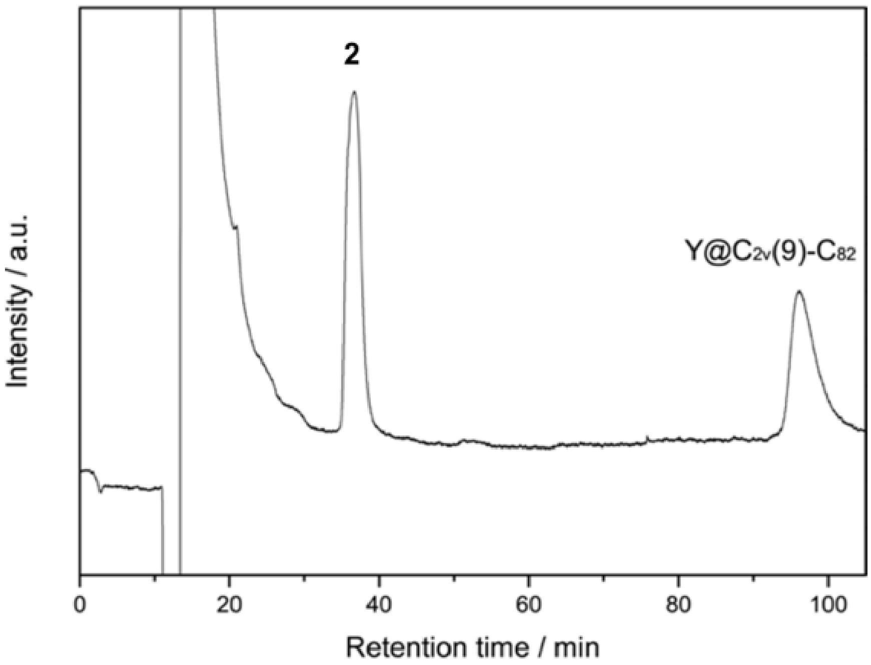

Figure S1. Preparative HPLC profiles of the reaction mixture of Y@C_{2v}(9)-C_{82} and N-arylbenzamidine. HPLC condition: 5PYE column (Φ20 mm × 250 mm); 20 mL injection volume; 10 mL/min toluene flow; room temperature; 330 nm detecting wavelength.

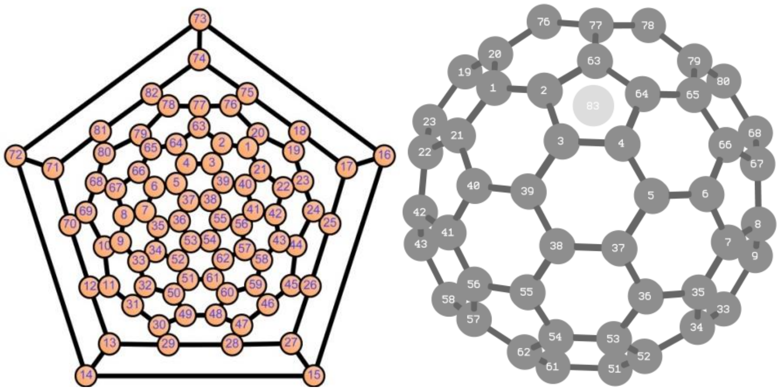

(A) (B)

Figure S2. Numbering of Y@C_{2v}(9)-C_{82}: (A) 2D view; (B) 3D view from front.

Table S1. Relative energies (R.E. in kcal/mol) of isomers of **2** predicated by HF level of theory.

Isomer	R.E.	Isomer	R.E.	Isomer	R.E.	Isomer	R.E.
(1,21)	13.9	(20,76)	3.6	(42,22)	8.1	(57,56)	20.0
(2, 1)	22.3	(21,22)	22.8	(42,43)	27.7	(58,57)	37.7
(2,63)	23.0	(21,40)	22.1	(51,61)	54.9	(58,43)	47.6
(3, 2)	24.0	(22,23)	23.6	(54,53)	17.0	(61,62)	33.3
(3,39)	30.5	(38,37)	40.5	(54,55)	9.5	(62,57)	22.7
(4, 3)	18.7	(39,38)	64.5	(54,62)	31.5	(63,77)	2.8
(19,23)	35.6	(40,39)	12.8	(55,38)	16.0	(75,76)	20.0
(20,19)	9.8	(41,40)	16.8	(56,55)	33.5	(76,77)	15.7
(20, 1)	31.0	(41,42)	13.0	(56,41)	0.0		

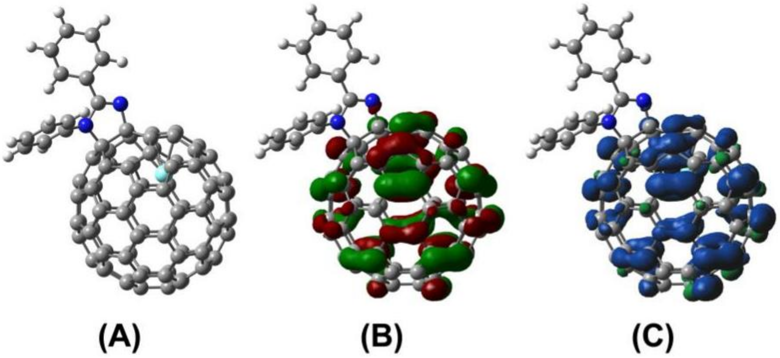

Figure S3. (A) Geometry, (B) SOMO and (C) spin density of isomer (20, 76) predicated by B3LYP method.

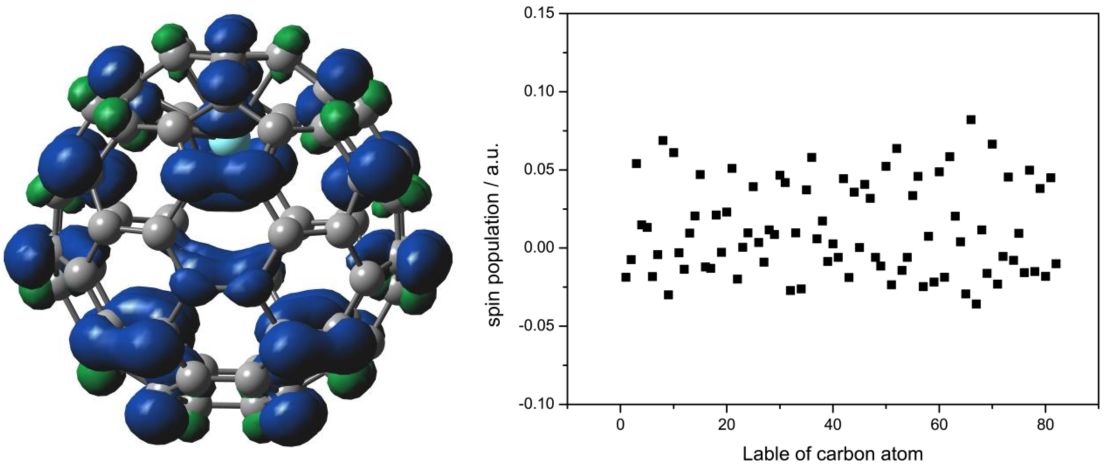

(A) (B)

Figure S4. Spin distribution of Y@C_{2v}(9)-C_{82}: (A) spin density and (B) spin population on carbon atoms

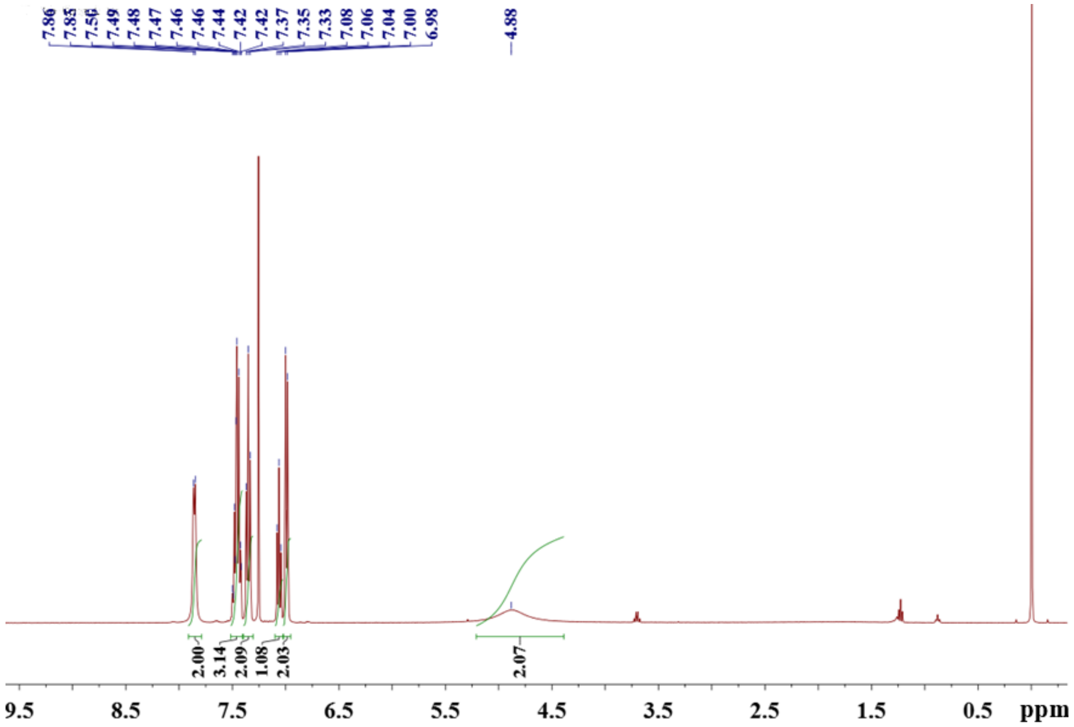

Figure S5. ^{1}H NMR of N-arylbenzamidine (**1**).
